# Effects of a flexibly delivered group-based acceptance and commitment therapy on reducing stress and enhancing psychological wellbeing in parents of school-age children during the COVID-19 pandemic: a quasi-experimental study

**DOI:** 10.3389/fpubh.2024.1485836

**Published:** 2024-12-12

**Authors:** Yim-Wah Mak, Doris Y. P. Leung, Xuelin Zhang, Joyce O. K. Chung, Petsy S. Y. Chow, Jiayin Ruan, Jerry W. F. Yeung

**Affiliations:** ^1^School of Nursing, The Hong Kong Polytechnic University, Kowloon, Hong Kong SAR, China; ^2^Mental Health and Development, Christian Family Service Centre, Hong Kong, Hong Kong SAR, China

**Keywords:** acceptance and commitment therapy, COVID-19, psychological distress, parental stress, wellbeing, real-world settings

## Abstract

**Introduction:**

COVID-19 has increased parental stress and significantly impacted the psychological well-being of individuals, especially parents of school-age children. Acceptance and Commitment Therapy posits that individuals can accept their unchangeable inner experiences (thoughts and feelings) while acting in ways aligned with their personal values, demonstrating effectiveness in reducing stress and improving psychological well-being, especially among parents of children with chronic illness. This study aimed to test the effectiveness of a group-based ACT, delivered flexibly, in improving stress and psychological well-being in parents with school-age children, regardless of their children’s chronic conditions, within a real-world context.

**Methods:**

This quasi-experimental study recruited parents with school-age children, through convenience sampling from the Christian Family Service Centre in Hong Kong. The group-based Acceptance and Commitment Therapy intervention consisted of five weekly sessions delivered via face-to-face meetings and online videoconferencing, consistent with participants’ preferences and COVID-19 related restrictions in Hong Kong. Paired t-tests and Generalized Estimating Equations following the intention-to-treat principle were used to examine the overall effects and the moderating effects of delivery methods.

**Results:**

The study included 250 parents, with an average age of 40.90 years, mostly women. The average age of their youngest child was 7.68 years. In line with COVID-19-related restrictions and participants’ preferences, 109 parents participated in online sessions, while 141 attended face-to-face sessions. Results showed significant small-to-medium improvements in various outcome variables after the intervention, including parental stress (Cohen’s *d* = 0.26), general stress (*d* = 0.18), depressive symptoms (*d* = 0.18), mental health literacy (*d* = 0.43), subjective wellbeing (*d* = 0.25), and psychological flexibility (*d* = 0.28). Notably, the online videoconferencing format was found to be as effective as the face-to-face format, with similar session attendance rates.

**Conclusion:**

The findings highlight the potential of group-based Acceptance and Commitment Therapy to alleviate stress and improve psychological well-being in parents of school-age children, regardless of the delivery method, especially during crises such as the COVID-19 pandemic. However, due to limitations in the study design, caution is warranted when interpreting the overall effects of group-based ACT on parent outcomes and the moderating role of delivery methods. Further research is needed to validate these findings and explore the nuances of delivery methods in similar real-world situations.

## 1 Introduction

Parental stress is a global phenomenon characterized by an aversive psychological response to the demands of raising and nurturing children (1). Even before the outbreak of the COVID-19 pandemic, the prevalence rates of parental stress were high (2–4), reaching up to 75% in Hong Kong, 69% in the United States (US), and 68% in United Kingdom (UK) (2–4). The onset of the pandemic in early 2020 exacerbated these stressors (5, 6). The World Health Organization (WHO) declared COVID-19 a “public health emergency of international concern” in January 2020 (7) and was later classified as a pandemic in March 2020 (8). This unprecedented crisis has profoundly impacted societies around the world, including Hong Kong, which reported its first case on January 23, 2020 (9) and faced five COVID-19 waves by December 31, 2022, each characterized by substantial increases in cases and deaths (10). To slow the spread of the virus, the government implemented measures such as lockdowns, stay-at-home orders, and social distancing (10). Schools closed intermittently and shifted to online learning. These closures, coupled with the rapid shift to remote work, economic uncertainty, and severe social restrictions, have intensified parental stress (11–13).

COVID-19 has forced parents of school-aged children to assume dual care and education responsibilities. This situation is further complicated by concerns about resource adequacy (1, 14), widespread uncertainty (15), and increased demands for homeschooling and employment (16). Previous evidence indicates that chronic stress and exhaustion can impair parents’ emotional awareness and responsiveness, affecting children’s development (17–20). Struggles to effectively fulfill parenting responsibilities may exacerbate feelings of low mood and inadequacy, especially given social pressures for “ideal parenting” (21, 22). Feeling unable to control stressful events may further impact parents’ mental health and reduce wellbeing (23), and the increased psychological burden of COVID-19 has led to a general increase in anxiety and depression (5, 24). Parental stress also significantly affects children and may contribute to the development of child psychopathology (1), impair their ability to regulate emotion (25), and develop behavioral problems over time (26–28). Given the profound impact of parental stress on parents and children, it is critical that resources are available to help parents of school-aged children manage and cope with these challenges, especially during difficult times like these.

Acceptance and commitment therapy (ACT) has emerged as a promising approach to support parents facing the significant challenges in Hong Kong (29) and during the COVID-19 pandemic (30, 31). Steaming from cognitive-behavioral tradition and grounded in the robust theoretical foundation, ACT focuses on acceptance of uncomfortable thoughts and feelings while emphasizing present-moment awareness and behavior change aligned with individual values. This emphasis on enhancing psychological flexibility, rather than solely targeting pathology, makes ACT particularly relevant for parents navigating the challenges during the COVID-19 pandemic. Meta-analytic evidence strongly supports the effectiveness of ACT in reducing parental stress (30–33), general stress (30), anxiety (30–33), depression (30–33), and improving subjective wellbeing (33) across various contexts (30–33).

However, existing studies have primarily focused on parents of children with chronic conditions, such as autism, asthma, or developmental delays (30), supporting parents to manage condition-specific psychological challenges. There is a noticeable gap specifically targeting the broader parenting challenges faced by everyday parents of school-age children, particularly intensified during the pandemic. With increased psychological difficulties potentially impacting parental responsiveness, expanding ACT interventions to this broader group of parents is critical to enhance stress management and support healthier caregiving environments, while also ensuring that these interventions are accessible and available in this challenging real-world context.

The expansion of remote mental health services has been essential for improving access to mental health care (34). Many healthcare providers have rapidly adopted online platforms like Zoom and Skype to maintain availability and accessibility (35). The National Alliance on Mental Illness has also offered both face-to-face and online options to support mental health in response to the pandemic (36). Organizations such as the WHO have provided self-help manuals using ACT for managing general stress during COVID-19 (37), but there remains a need for more targeted support specifically for parents of school-age children. ACT providers have increasingly adapted to virtual platforms, studies have demonstrated similar efficacy between videoconferencing and face-to-face delivery in addressing parental stress in general parents (38), as well as in managing anxiety, depression, and wellbeing for patients with chronic pain (39). Therefore, the current study aimed to expand access to mental health services through both offline and online platforms for parents of school-age children during the COVID-19 pandemic.

The objectives of this study were to assess the overall effects of a group-based ACT parenting program in reducing perceived stress and improving mental health outcomes for parents with school-age children and evaluated the moderating effects of delivery formats on these outcomes. The primary hypothesis was that parent stress, psychological distress, mental health literacy, and subjective wellbeing would significantly improve after a group-based ACT parenting program delivered flexibility via either online videoconferencing or face-to-face in-person formats. The secondary hypothesis was that two delivery formats would have similar effects on these health outcomes.

## 2 Materials and methods

### 2.1 Study design and participants

This study employed a non-controlled clinical trial with a quasi-experimental design. Participants were recruited through convenience sampling from the Christian Family Service Centre (CFSC), a non-governmental organization providing services in the Kwun Tong, Wong Tai Sin, and Sai Kung districts of Kowloon East, Hong Kong. The study received approval from the Human Subjects Ethics Sub-Committee of The Hong Kong Polytechnic University, as well as from the participating organizations.

Participants were eligible if they were aged 18 or above, parents with at least one child studying in kindergartens, primary or secondary schools, regardless of the child’s health condition, living with the child in the same household, and able to speak Cantonese and understand Chinese writing. If parents had more than one school-age child, the youngest child was the index child for this study. The exclusion criteria were parents with severe psychological distress (i.e., depression score in Depression, Anxiety and Stress Scale (DASS) > 20 or anxiety score in DASS >14, or stress score in DASS >25) (40, 41), or currently receiving parent training programs in promoting psychological health.

### 2.2 ACT parenting program

The ACT parenting program protocol was informed by the previous study (42) and adopted ACT metaphors and exercises tailored for Chinese parents. The program consisted of five weekly sessions, each lasting 2 h and delivered in a group format of 4–8 parents. The group-based format can facilitate the normalization of parenting challenges and foster peer support among group members (43).

Initially, the ACT parenting program was offered in two formats: face-to-face and online videoconferencing, allowing parents to choose their preferred mode of participation. However, due to COVID-19-related restrictions in Hong Kong from January to March 2022, which limiting group gatherings, the program was conducted exclusively via online videoconferencing during this period, accommodating a total of 36 parents across five ACT groups.

The ACT parenting program covered six core elements of ACT (42), aiming to enhance parental psychological flexibility in navigating child-rearing situations, particularly during the COVID-19 pandemic. Specific objectives included enhancing present moment awareness and full attention in parenthood, fostering acceptance of stressful events or situations, creating a validating emotional atmosphere in parent–child interactions, improving interactions with private events by promoting the diffusion of thoughts, feelings, and emotions, and leading to actions directed at prioritizing parenting values. Throughout the sessions, ACT metaphors and experiential exercises were employed, supplemented by visual aids in PowerPoint slides and information sheets. All communication during ACT parenting sessions, between facilitator and group members, was synchronous. A detailed overview of the training program is presented in Table 1.

**Table 1:** ACT parenting program structure.

	Topics	ACT core element
*Session 1*: Accepting as it is	Introducing sources of parenting stress and adverse effects of excessive stress on parents and their children Preparing the participants for group interaction and therapy Engaging them into the mindset and enhance the acceptance of the difficulties and sufferings of parenthood Leading them from the paradox of control to hopelessness to a creative way out	Present moment and acceptance
*Session 2*: Fusion and defusion	Enhancing participants in finding their fusion in parenting Facilitating them in defusing their thoughts and feelings Helping them in exploring their parenting values	Defusion and chosen values
*Session 3*: Choice point	Exploring and clarifying parenting values of participants Facilitating them in prioritizing parenting values Enhancing them in applying their parenting values in real-life situations	Chosen values
*Session 4*: The observing self	Facilitating participants in experiencing the “observing self” Enhancing them in mindful of their judgments in parenting Helping them in applying the observing self in their daily parenting	Self-as-context
*Session 5*: Committed to upbringing	Facilitating participants in goals and committed aligned with their parenting values Enhancing their ability in mindfully planning and implementing ACT strategies	Committed action

ACT, acceptance and commitment therapy.

The implementation of the ACT intervention started in May 2021 and continued until December 2022, led by three facilitators. These facilitators, who were registered nurses or certified social workers with at least 2 years of experience in implementing psychological interventions, underwent training to deliver the ACT parenting program. Facilitator training included a three-day ACT workshop focused on ACT skills, followed by additional guidance sessions that featured demonstrations, return demonstrations, and discussions using the finalized intervention protocol of this study, led by the first author, an experienced ACT researcher and counselor. Before the implementation of the program, all facilitators underwent competency checks. Each group-based ACT session was then led by one single facilitator, who remained with the same group for all five sessions. Additionally, all ACT sessions were videotaped with parental consent, and facilitators reviewed the recordings after each session for questions and discussions. No deviations from the intervention protocol were noted.

### 2.3 Measures

Sociodemographic data were collected at baseline (T0). Outcome assessments conducted at baseline (T0) and immediately after the last session (T1), during June 2020 to December 2022.

#### 2.3.1 Socio-demographics

Socio-demographic data, include age, gender, educational level, marital status, number of children and their age, as well as whether had taken a course in stress management.

#### 2.3.2 Parental stress

Parental stress experienced when raising children was assessed by the Chinese version of the Parental Stress Scale (PSS) (44, 45), which consists of 17 items measuring two subscales of parental strain and lack of parental satisfaction, were rated on a 6-point Likert scale ranging from 1 (disagree very much) to 6 (agree very much). Overall scores and two subscale scores were calculated, with higher scores indicating higher levels of negative experience. The PSS has demonstrated acceptable psychometric properties (45). In this sample, acceptable internal consistencies of parental stress were obtained with Cronbach’s *α* of 0.88 at T0 and 0.87 at T1, parent strain (T0: α = 0.87; T1: α = 0.88) and lack of parental satisfaction (T0: α = 0.91; T1: α = 0.92).

#### 2.3.3 General psychological symptoms

The Chinese version of the Depression, Anxiety, and Stress Scale (DASS) (40, 41) was used to assess general psychological symptoms, specifically focusing on general stress, anxiety, and depression. The DASS is a 21-item self-report measure consisting of three 7-item subscales: the Stress Subscale (DASS-SS), the Anxiety Subscale (DASS-AS), and the Depressive Subscale (DASS-DS). Each subscale was rated on a 4-point Likert scale, with higher scores indicating greater severity of the respective symptom. The DASS subscales has demonstrated good reliability and validity for use in nonclinical settings in Hong Kong (39). In the present study, the DASS subscales showed acceptable internal consistency (*α*) at both time points, ranging from 0.60 to 0.78 at T0 and 0.81 to 0.86 at T1.

#### 2.3.4 Mental health literacy

Parent mental health literacy was assessed by the Mental Health Literacy Scale (MHLS) (46), which consists of 26 items rated on a 5-point Likert scale ranging from 1 (completely disagree) to 5 (completely agree). It comprises five subscales: maintenance of positive mental health, recognition of mental illness, attitude toward mental illness stigma, help-seeking efficacy, and help-seeking attitude. Scores of the scale and its’ subscales were computed with higher scores indicating higher levels of mental health literacy. The scale has demonstrated good construct validity and acceptable internal consistency (46). The Cronbach’s *α* values of overall MHLS scale were 0.83 at both T0 and 0.83 at T1, while the subscales ranged from 0.78 to 0.88 at T0 and 0.80 to 0.90 at T1 in the present sample.

#### 2.3.5 Subjective wellbeing

Subjective wellbeing was measured using the World Health Organization Wellbeing Index (WHO-5), a widely used brief standard global rating scale (47–49). The WHO-5 consists of 5 items with responses scored on a 6-point Likert scale ranging from 0 (at no time) to 5 (all of the time). The reporting period is 2 weeks. The range of the total raw scores is 0–25, which were then multiplied by 4 to produce the final score, with higher scores indicating better wellbeing. The WHO-5 has shown to have high clinometric validity (50), as it can be used in many different settings, irrespective of the presence or absence of comorbid conditions. In this sample, the WHO-5 index showed excellent internal consistency in WHO-5 (T0: *α* = 0.94; T1: *α* = 0.95).

#### 2.3.6 Psychological flexibility

The 15-item Chinese version of the Brief Experiential Avoidance Questionnaire (BEAQ) measures parents’ psychological flexibility to accept undesirable thoughts and feelings while acting in congruence with personal values and goals (51, 52). The items are rated on a 6-point Likert scale ranging from 1 (definitely disagree) to 6 (definitely agree). The total BEAQ score ranges from 15 to 90. The higher the score, the lower degree of psychological flexibility. The Chinese version of BEAQ has been shown to have acceptable internal consistency (*α* >0.78) and test–retest reliability over 2 months (52). In the sample of this study, the BEAQ total score showed good internal consistency (T0: Cronbach’s *α* = 0.82; T1: Cronbach’s *α* = 0.86).

### 2.4 Statistical analysis

Power analysis using GLIMMPSE software (53) was conducted to determine the sample size for detecting a small effect size (0.2) with 80% power, an alpha of 0.05, and a correlation of 0.5 between two repeated measures. To allow for an estimated 20% incompletion rate due to missing data or attrition, a recruitment target of 250 parents was set.

Descriptive statistics were employed to summarize sociodemographic and baseline variables. The Shapiro–Wilk test confirmed the normality of continuous data, including scores for overall parental stress and its subscales, anxiety, depression, general stress, mental health literacy and its subscales, as well as subjective wellbeing and psychological flexibility. Baseline differences between the online videoconferencing and face-to-face groups were assessed using Chi-Square tests for categorical variables and independent t-tests for continuous variables.

Changes in outcomes from baseline (T0) to post-intervention (T1) were examined with paired-sample *t*-tests. Generalized Estimating Equations (GEEs) with a first-order autoregressive (AR[1]) covariance structure were used to assess the intervention’s effects on parental stress, general stress, anxiety, depression, mental health literacy, subjective wellbeing, and psychological flexibility. The models adjusted for potential confounders, including sociodemographic factors, delivery mode, and COVID-19 related restrictions (e.g., public gathering bans, school closure), as indicated in previous research (11–13, 54–56). Predictive margins and standard errors (SE) for each outcome were calculated.

To evaluate the moderating role of delivery modes (face-to-face vs. online), GEE models were employed, incorporating an interaction term (group-by-time) adjusted for sociodemographic factors and COVID-19 related restrictions. A significant interaction effect would indicate differing intervention effects by delivery format. GEEs were selected for their ability to handle missing data under the assumption of randomness, facilitating an intention-to-treat analysis without the need for imputation (57).

Within-group Cohen’s d effect sizes were calculated to assess the magnitude of the effects of the intervention, with thresholds of 0.20, 0.50, and 0.80 indicating small, medium, and large effects, respectively (58). All analyses were conducted using SPSS, Version 28.0, with a significance level set at *p* < 0.05.

### 2.5 Procedure of ACT parenting program delivery

Between June 2020 and December 2022, we approached 1,400 potential participants by sending invitation emails with project promotion letters, enrollment, and screening forms through our CFSC network. This included outreach to 110 schools (from kindergartens to secondary schools), 181 non-governmental organizations (NGOs), and 123 community churches. CFSC also conducted introductory talks to schools and NGOs to encourage participation.

Of those approached, 454 parents (32.43%) submitted enrollment and screening forms and were contacted by CFSC staff for further screening. Among these, 286 parents (63.00%) continued with the process, while 168 (37.00%) were excluded due to various reasons: declining participation (*n* = 27), scheduling conflicts (*n* = 96), lost contact (*n* = 4), or referral for more intensive treatment due to elevated DASS scores (*n* = 41). Ultimately, 250 parents (250/286, 87.41%) provided oral and written consent to participate in the ACT parenting program and completed baseline assessment.

All 250 participants engaged in the group-based ACT parenting program, which included five weekly, two-hour sessions, available in face-to-face and online videoconferencing formats. Of these, 109 (43.60%) attended in person, while 141 (56.40%) participated in online videoconferencing via Zoom.

Furthermore, parents reported practical reasons for missed sessions or dropout, including illness, childcare issues, and sudden stressful events, such as sporadic changes in social distancing policies. Despite these challenges, most participants attended the sessions, resulting in low losses to follow-up and no serious adverse effects reported. On average, participants attended 4.29 out of 5 sessions (SD = 1.04). Specifically, parents in the face-to-face group attended an average of 4.40 sessions (SD = 0.96), while those in the online group attended 4.20 sessions (SD = 1.10), with no significant difference in attendance between the groups (*t* = 1.54, *p* = 0.120). Post-intervention assessment data were missing for 20 parents (8%), including 5 from the face-to-face group and 15 from the online group. The difference in missing data was not statistically significant between the groups (χ^2^ = 3.06; *p* = 0.080).

## 3 Results

A total of 250 parents participated in this study, with a mean age of 40.90 years (SD = 6.29), ranging from 28 to 65 years. Among these participants, 232 (92.80%) were mothers, the majority were married (88.80%), and 138 (55.20%) had an education level of secondary school or below. On average, parents had 1.70 children (SD = 0.66), with the number of children ranging from 1 to 4. The mean age of the youngest child was 7.68 years (SD = 3.87), ranging from 1 month to 26 years, while the overall average age of the children was 9.05 years (SD = 4.20), with ages ranging from 0.1 to 29.5 years. For the oldest child in each family, the mean age was 10.50 years (SD = 5.31), with a range of 1 to 33 years. Additionally, 33 parents (13.20%) reported having prior training in stress management. In addition, 25 parents (10.00%) were enrolled during full-day school closures, 161 (64.40%) during partial-day school closures, and 64 (25.60%) during periods with no school closure.

The comparisons of sociodemographic characteristics and baseline variables between the face-to-face and online videoconferencing groups are presented in Table 2. Notable differences were found between the two groups regarding certain sociodemographic characteristics and outcome variables. In the online videoconferencing group, a higher percentage of parents held tertiary education or above (53.68%) compared to the face-to-face group (29.25%) (*p* < 0.001). Additionally, parents in the online group had children with a younger average age (Mean = 8.34 years) than those in the face-to-face group (M = 9.97 years, *p* = 0.003), and the oldest child in the online group was also younger on average (M = 9.54 years) compared to the face-to-face group (M = 11.81 years, *p* < 0.001). Furthermore, a greater proportion of parents in the online group had previously received stress management training (19.71% vs. 5.61%, *p* = 0.001). Regarding baseline outcome variables, the online videoconferencing group reported higher total parental stress (M = 54.75) than the face-to-face group (M = 49.86) (*p* = 0.039). They also showed greater anxiety levels (M = 6.63 vs. 5.72, *p* = 0.012) and a more negative attitude toward stigma (M = 16.26 vs. 15.38, *p* = 0.019). No significant differences were observed between the groups for other factors, including the subscales of parental stress, depression, general stress, subjective wellbeing, psychological flexibility, and other subscales of mental health literacy, such as help-seeking efficacy and symptom recognition.

**Table 2:** Social-demographic characteristics of participating parents and their reported baseline outcome variables.

Subject characteristics	Face-to-face group (*n* = 109)	Online videoconferencing group (*n* = 141)	*x*^2^/*t*	*p*-value
	M(SD) or f(%)	M(SD) or f (%)		
Social demographic characteristics				
Age (years)	40.92 (6.24)	40.88(6.34)	0.046	0.873
Number of children	1.77 (0.68)	1.64 (0.64)	1.589	0.652
Age of youngest child	8.25 (3.97)	7.25 (3.74)	2.002	0.066
Age of oldest child	11.81 (5.71)	9.54 (4.62)	3.637	**<0.001**
Average age of children	9.97 (4.46)	8.34 (3.87)	3.022	**0.003**
Gender				
Male	5 (4.59%)	13 (9.22%)	1.60	0.218
Female	104 (95.41%)	128 (90.78%)		
Marital status				
Married	98 (90.74%)	124 (91.18%)	0.014	0.906
Unmarried/Widowed/Divorced/separated separated/devoice	10 (9.26%)	12 (8.82%)		
Education attainment				
Primary education or below	3 (2.83%)	12 (8.82%)	22.575 **< 0.001**	
Secondary education	72 (67.92%)	51 (37.50%)		
Tertiary education or above	31 (29.25%)	73 (53.68%)		
Previous stress training				
Yes	6 (5.61%)	27 (19.71%)	10.213	**0.001**
No	101 (94.39%)	110 (80.29%)		
COVID-19 related school closure				
Full-day school closure	0	25 (17.73%)	21.827	**<0.001**
Partial-day school closure	76 (69.72%)	85 (60.28%)		
No closure	33 (30.28%)	31 (21.99%)		
Baseline outcome variables				
Parental stress (PSS)				
Parental strain	35.95 (9.06)	38.07 (8.26)	−1.898	0.176
Lack of parental satisfaction	13.95 (5.10)	16.76 (5.43)	−4.141	0.537
Total score	49.86 (12.54)	54.75(10.19)	−3.362	**0.039**
General psychological symptoms (DASS)				
General stress (DASS-SS)	11.52 (5.91)	13.61(6.91)	−2.516	0.175
Anxiety (DASS-AS)	5.72 (3.72)	6.63(4.43)	−1.717	**0.012**
Depression (DASS-DS)	6.11 (4.32)	6.62 (4.85)	−0.860	0.101
Mental health literacy (MHLS)				
Maintenance	39.22 (5.80)	38.37 (6.56)	1.055	0.315
Recognition	16.85 (2.24)	16.91 (2.63)	−0.176	0.602
Attitude toward stigma	15.38 (3.44)	16.26 (4.20)	−1.795	**0.019**
Help seeking efficacy	9.92 (2.42)	10.40 (2.44)	−1.558	0.490
Help seeking attitude	11.38 (2.33)	10.64 (2.36)	2.433	0.539
Total score	97.86 (8.95)	96.04 (11.36)	1.346	0.105
Subjective wellbeing (WHO-5)	55.26 (22.27)	48.46(19.98)	2.523	0.109
Psychological flexibility (BEAQ)	53.78 (8.81)	52.40 (9.15)	1.202	0.545

BEAQ, Brief Experiential Avoidance Questionnaire; DASS, Depression, Anxiety, and Stress Scale; DASS-AS, Depression, Anxiety, and Stress Scale- Anxiety Subscale; DASS-DS, Depression, Anxiety, and Stress Scale- Depressive Subscale; DASS-SS, Depression, Anxiety, and Stress Scale- Stress Subscale; f, Frequency; M, mean; MHLS, Mental Health Literacy Scale; n, total number of participants; PSS, Parental Stress Scale; SD, Standard deviation; t, t-statistics; WHO-5, World Health Organization Wellbeing Index; χ^2^, Chi-square. *P* values in bold are statistically significant.

The changes in outcome measures for parents from pre-intervention to post-intervention in both groups are presented in Table 3. Paired T-tests indicated that most outcomes showed significant improvements following the ACT parenting sessions at T1; however, no significant changes were observed in anxiety (*p* = 0.755) and symptom recognition within mental health literacy (*p* = 0.127). Specifically, parental stress showed significant reductions in parental strain, lack of parental satisfaction, and total scores on the Parental Stress Scale (PSS) at T1, all with small effect sizes (*p*s: < 0.001–0.008; Cohen’s *d*: 0.18–0.26). Additionally, significant decreases were noted in general stress and depression symptoms (*p*s: 0.007–0.008; *d* = 0.18). Notably, overall mental health literacy and four out of five mental health literacy subscales exhibited significant increases at T1 (*p*s: < 0.001–0.023; *d*: 0.15–0.51), alongside improvements in subjective wellbeing (*p* < 0.001; d = 0.25) and psychological flexibility (*p* < 0.001; *d* = 0.28). Most of these intervention effects remained significant in the Generalized Estimating Equations (GEEs) analysis, with the exception of the help-seeking attitude subscale (*p* = 0.078).

**Table 3:** Changes in outcome variables from pre-intervention to post-intervention.

Outcome variables	Unadjusted				Adjusted models		
	T0 [*M (SD)*]	T1 [*M (SD)*]	Paired *t* test (*p*)^a^	Cohen’s *d*	T0 [*M (SE)*]^b^	T1 [*M (SE)*]^b^	GEE *β* (*p*)^b^
Parental stress (PSS)							
Parental strain	37.19 (8.55)	35.78 (8.78)	**2.670 (0.008)**	0.18	37.13 (0.57)	35.60 (0.59)	**−1.54 (0.005)**
Lack of parental satisfaction	15.53 (5.39)	14.71 (5.34)	**3.271 (0.001)**	0.22	15.44 (0.35)	14.70 (0.36)	**−0.74 (0.004)**
Total score	52.68 (11.25)	50.47 (10.97)	**3.914 (< 0.001)**	0.26	52.53 (0.74)	50.29 (0.73)	**−2.24 (< 0.001)**
General psychological symptoms (DASS)							
General Stress (DASS-SS)	12.74 (6.50)	11.50 (7.80)	**2.681 (0.008)**	0.18	12.73 (0.43)	11.54 (0.53)	**−1.20 (0.012)**
Anxiety (DASS-AS)	6.24 (4.17)	6.36 (5.91)	−0.312 (0.755)	0.02	6.21 (0.27)	6.40 (0.40)	0.19 (0.614)
Depression (DASS-DS)	6.37 (4.61)	5.27 (6.19)	**2.733 (0.007)**	0.18	6.40 (0.30)	5.30 (0.42)	**−1.10 (0.008)**
Mental health literacy (MHLS)							
Maintenance	38.65 (6.26)	41.64 (5.40)	**−7.570 (< 0.001)**	0.51	38.57 (0.41)	41.546 (0.37)	**2.97 (< 0.001)**
Recognition	16.86 (2.47)	16.58 (2.53)	1.532 (0.127)	0.10	16.90 (0.16)	16.60 (0.17)	−0.31 (0.105)
Attitude toward stigma	15.84 (3.87)	14.51 (4.14)	**5.124 (< 0.001)**	0.34	16.00 (0.26)	14.62 (0.28)	**−1.37 (< 0.001)**
Help seeking efficacy	10.18 (2.43)	11.42 (2.50)	**−6.986 (< 0.001)**	0.46	10.18 (0.16)	11.40 (0.17)	**1.21 (< 0.001)**
Help seeking attitude	10.89 (2.38)	11.30 (2.45)	**−2.288 (0.023)**	0.15	10.96 (0.16)	11.27 (0.17)	0.31 (0.078)
Total score	96.71 (10.57)	101.62 (11.19)	**−6.470 (< 0.001)**	0.43	96.64 (0.69)	101.41 (0.75)	**4.76 (< 0.001)**
Subjective wellbeing (WHO-5)	51.90 (20.98)	57.34 (22.87)	**−3,770 (< 0.001)**	0.25	51.44 (1.39)	57.03 (1.53)	**5.59 (< 0.001)**
Psychological flexibility (BEAQ)	52.89 (9.04)	50.09 (10.44)	**4.240 (< 0.001)**	0.28	53.19 (0.59)	50.23 (0.69)	**−2.97 (< 0.001)**

β, Unstandardized value based on Generalized estimating equation models; BEAQ, Brief Experiential Avoidance Questionnaire; DASS, Depression, Anxiety, and Stress Scale; DASS-AS, Depression, Anxiety, and Stress Scale- Anxiety Subscale; DASS-DS, Depression, Anxiety, and Stress Scale- Depressive Subscale; DASS-SS, Depression, Anxiety, and Stress Scale- Stress Subscale; M, Mean; MHLS, Mental Health Literacy Scale; PSS, Parental Stress Scale; SD, Standard deviation; SE, Standard error; T0, Timepoint baseline; T1, Timepoint post-intervention; WHO-5, World Health Organization Wellbeing Index.

a *t-value and p-value for the paired t-test.*

b Social-demographic factors, including age, gender, educational level, number of children, age of the youngest child, average age of children, marital status, stress management training experience, delivery mode and COVID-19 related restrictions were controlled in GEE models. *P* values in bold are statistically significant.

The results of the moderating effects of delivery mode on those outcomes, after accounting for sociodemographic factors and COVID-19-related restrictions, are summarized in Table 4. The online videoconferencing group showed a promising improvement in the lack of parental satisfaction subscale score compared to the face-to-face group (*β* = −1.05, 95% CI [−2.10, 0.05]) post-intervention, although this was not statistically significant (*p* = 0.051). Overall, there were no significant differences observed between the face-to-face and online videoconferencing groups for these health outcomes post-intervention, indicating that both delivery modes were effective.

**Table 4:** Comparing the outcome variables between delivery mode across time points.

Outcome variable	Face-to-face group		Online videoconferencing group	GEE model^a^			
	T0 [*M (SD)*]	T1 [*M (SD)*]	T0 [*M (SD)*]	T1 [*M (SD)*]	*β* (group × time)^a^	95% CI^a^	*P* ^a^
Parental stress (PSS)							
Parental strain	35.95 (9.06)	35.07 (8.26)	38.07 (8.26)	36.35 (9.12)	−0.84	−2.98, 1.31	0.445
Lack of parental satisfaction	13.95 (5.10)	13.66 (4.94)	16.76 (5.43)	15.48 (5.52)	−1.05	−2.10, 0.05	0.051
Total score	49.86 (12.54)	48.73 (10.90)	54.75 (10.19)	51.84 (10.74)	−1.87	−4.32, 0.57	0.133
General psychological symptoms (DASS)							
General Stress (DASS-SS)	11.52 (5.91)	10.35 (6.30)	13.61 (6.91)	12.53 (8.70)	−0.12	−2.00, 1.77	0.904
Anxiety (DASS-AS)	5.72 (3.72)	5.96 (5.07)	6.63 (4.43)	6.66 (6.47)	−0.43	−1.87, 1.01	0.557
Depression (DASS-DS)	6.11 (4.32)	4.58 (4.82)	6.62 (4.85)	5.86 (7.07)	0.62	−0.95, 2.19	0.438
Mental health literacy (MHLS)							
Maintenance	39.22 (5.80)	41.50 (4.91)	38.37 (6.56)	41.65 (5.70)	0.77	−0.78 2.31	0.330
Recognition	16.85 (2.24)	16.25 (2.32)	16.91 (2.63)	16.87 (2.65)	0.54	−0.19, 1.28	0.149
Attitude toward stigma	15.38 (3.44)	14.02 (3.68)	16.28 (4.20)	15.00 (4.48)	−0.12	−1.15, 0.91	0.821
Help seeking efficacy	9.92 (2.42)	11.19 (2.32)	10.40 (2.44)	11.52 (2.70)	−0.17	−0.88, 0.53	0.626
Help seeking attitude	11.38 (2.33)	11.60 (2.23)	10.64 (2.36)	11.08 (2.61)	0.19	−0.52, 0.88	0.601
Total score	97.86 (8.95)	102.52 (9.47)	96.04 (11.36)	100.82 (12.22)	0.08	−2.80, 2.96	0.956
Subjective wellbeing (WHO-5)	55.26 (22.27)	60.07 (2.26)	48.46 (19.97)	54.67 (2.07)	0.22	−6.19, 5.76	0.942
Psychological flexibility (BEAQ)	53.78 (8.81)	50.96 (9.31)	52.40 (9.15)	49.37 (11.34)	−0.59	−3.16, 1.97	0.651

β, Unstandardized value based on the models; BEAQ, Brief Experiential Avoidance Questionnaire; DASS, Depression, Anxiety, and Stress Scale; DASS-AS, Depression, Anxiety, and Stress Scale- Anxiety Subscale; DASS-DS, Depression, Anxiety, and Stress Scale- Depressive Subscale; DASS-SS, Depression, Anxiety, and Stress Scale- Stress Subscale; GEE, Generalized estimating equation; M, Mean; MHLS, Mental Health Literacy Scale; PSS, Parental Stress Scale; SD, Standard deviation; T0, Timepoint baseline; T1, Timepoint post-intervention; WHO-5, World Health Organization Wellbeing Index.

a Social-demographic factors, including age, gender, educational level, number of children, age of the youngest child, average age of children, marital status, stress management training experience and COVID-19 related restrictions were controlled in the models.

## 4 Discussion

This study examined the effects of a flexibly delivered group-based ACT parenting program for parents with school-age children in Hong Kong during the COVID-19 pandemic. The results indicate that the program, delivered through either face-to-face or online videoconferencing formats, significantly reduced parental stress, general stress, and depression while also improving mental health literacy and subjective wellbeing post-intervention. However, the program did not produce significant effects on anxiety levels after the intervention, resulting in partial support for the primary hypothesis. Additionally, no significant moderating effect of delivery mode was found on these outcomes post-intervention, with intervention effects being similar across both groups for all measures, thus fully supporting the secondary hypothesis.

The findings from this study are well-aligned with the current literature on the benefits of ACT intervention on parental stress, general stress, depression symptoms and subjective wellbeing subjective wellbeing for parents (30–33), although these studies particularly focused on parents of those with chronical ill children. This study extends previous findings by demonstrating positive significant effects of the ACT parenting program on parents of school-age children in a real-world setting without controlling for the recruitment of the children with specific diagnosed conditions. While previous meta-analyses of parents with chronic illness indicated medium-sized effects of ACT on depression and subjective wellbeing (33), the effect sizes in this study were smaller. This difference might be attributed to the relatively lower baseline levels of depression and higher levels of subjective wellbeing among the participants in this study compared to parents with clinical concerns. For instance, the mean baseline depression score (*M* = 6.40, *SD* = 4.62) was within the normal range on the DASS-DS. The subjective wellbeing of participants in this study was relatively higher compared to parents of children with attention-deficit/hyperactivity disorder during the COVID-19 pandemic (59). It is possible that participants in this study may have had less room for larger improvements in these areas.

The ACT parenting program showed particularly valuable during the COVID-19 pandemic, which brought unprecedented stress and uncertainty to families, helped parents increase their awareness of their own emotional reactions and better manage their psychological challenges (60). Importantly, the strategies learned through ACT allowed parents to effectively adapt to the challenges of their role as parents, encouraging them to be more present and supportive figures for their children (61), fostering positive parent–child interactions and effective engagement in parenthood, which may further promote parental mental health and wellbeing over time. Significant positive effect in psychological flexibility, along with the significant improvements in these health outcomes, suggest that the ACT parenting program can be a valuable resource for supporting parents during challenging times.

While the goal of ACT is not to directly reduce psychological symptoms like anxiety, but rather to promote greater psychological flexibility, with improvements in symptoms considered desirable side effects (42). This study found a non-significant difference in anxiety levels after ACT parenting program, with a slight increase in DASS-AS score observed. This pattern contrasts with findings from earlier ACT parenting studies (32, 33, 62). It is possible that the program helped parents become more aware of their anxiety, initially leading to higher self-reported anxiety levels as they became more conscious of their internal experiences. This heightened awareness may have occurred before they fully integrated the coping strategies taught in ACT. In addition, the timing of the post-intervention assessment may have played a role. For some parents, the intervention sessions took place during stable periods of the pandemic, but lockdown measures were implemented afterwards, during the post-intervention assessment period. This potential increase in anxiety levels (63) and may have overshadowed the potential effects of the ACT parenting program on anxiety.

This study is the first to examine the effects of a group-based ACT parenting program on mental health literacy in parents. The results showed significant improvements with small-to-moderate-size in overall mental health literacy, primarily driven by positive changes across three out of five subscales: symptom maintenance, attitudes toward stigma, and help-seeking efficacy. The program’s focus on enhancing psychological flexibility may have empowered parents to become more aware of their psychological distress related to their own knowledge deficits. Instead of focusing on reducing distress, they were encouraged to take action aligned with their identified values. This may have led them to actively seek out mental health information and enhance their efficacy in using preventative mental health knowledge. Furthermore, the program’s use of metaphors and experiential learning approaches, rather than didactic psychoeducation, likely facilitated a deeper understanding of mental health concepts through their own experiences or those of their group members (64). Additionally, exposure to individuals sharing their stories of psychological distress during the ACT sessions could have helped reduce stigma attitudes (65). However, the lack of significant improvement in the help-seeking attitude subscale after the intervention suggests that sociodemographic factors, COVID-19-related restrictions, and delivery mode may have a greater influence on shaping help-seeking attitudes than the group-based ACT parenting program itself. While the program effectively addressed parental challenges and coping strategies during the pandemic, it did not specifically target beliefs and attitudes regarding help-seeking. Personal characteristics, along with the pandemic context, such as access to services, societal attitudes toward mental health, and healthcare affordability, may have further influenced these outcomes (66).

This study found relatively stable scores in the symptom recognition subscale of the mental health literacy measure post-intervention, with no statistically significant difference observed with or without adjustment. This finding may be explained by the relatively limited diversity of symptoms observed in the group environment. Participants in this study experienced low levels of psychological symptoms at baseline, with mean scores on anxiety and depression falling within the normal range (Anxiety measured by DASS-AS: M = 6.23, SD = 4.15; Depression measured by DASS-DS: M = 6.40, SD = 4.62). Since participants were not heavily exposed to significant psychological symptoms, the ACT sessions provided limited opportunities for them to observe and learn about psychological symptoms through shared experiences within the group setting. Another possible explanation for the stable scores on the symptom recognition subscale could be the heightened focus on acceptance of distressing thoughts and emotions. This focus might have led participants to interpret symptoms in a more nuanced way, recognizing them as part of a broader range of normal human experiences rather than pathological. As a result, participants may have reported lower recognition of symptoms because they were less likely to categorize their experiences as indicative of psychological disorders.

The moderating results indicated that the effects of group-based ACT did not differ by delivery modality on all health outcomes post-intervention after adjustment. Both face-to-face and online formats effectively increased parents’ self-awareness and practical functioning by contextualizing individual experiences within personal values and accepting both negative and positive experiences, yielding similar effects on these health outcomes among parents with school-age children. These findings align with previous research (38, 39, 67), supporting the flexibility of the program.

Notably, the online videoconferencing group showed a promising improvement in parental satisfaction, as measured by the parental stress subscale, post-intervention after adjusting for parents’ sociodemographic characteristics and COVID-19-related restrictions. This enhanced parental satisfaction may be attributed to some advantages of the online format, allowing parents to engage more easily from home, thereby reducing scheduling and childcare challenges. The anonymity of online participation may also foster a more open engagement, leading to a more positive parenting experience (68). Furthermore, the online format was found to be equally feasible and acceptable to parents, with similar rates of group attendance and dropout compared to the face-to-face format. These results suggest that delivering ACT interventions remotely through online platforms can be an effective alternative during circumstances such as the COVID-19 pandemic. However, it is crucial to recognize the non-equivalence of the group design, which highlights important differences between the two groups. Parents in the online group had a higher percentage of tertiary education, younger average age of children, a younger oldest child, a greater proportion with prior stress management training and reported elevated levels of baseline total parental stress, anxiety, and stigma toward mental health. These findings suggest that the accessibility of online formats may be more appealing to individuals with existing mental health challenges, potentially impacting both their participation and the overall efficacy of the intervention. Therefore, interpretations of these results should be made with caution.

## 5 Limitations and future directions

Overall, while the findings are promising, several methodological limitations warrant cautious interpretation of the positive effects. First, the absence of a control group raises questions about whether the observed outcomes can be attributed specifically to the ACT intervention or if they are influenced by other factors. Additionally, the non-random assignment of participants to the face-to-face and online videoconferencing groups introduces pre-existing differences that may have affected the significant differences in outcomes. To rigorously compare the efficacy of the two delivery formats, a randomized controlled trial is essential. Moreover, the lack of baseline data on children’s health status restricts the ability to explore potential moderating effects. The predominantly female sample and lack of representativeness among all Hong Kong parents further limit the generalizability of the findings to broader populations and diverse cultural contexts. Future research should aim to include more diverse samples to enhance the applicability of the results. Furthermore, the absence of long-term follow-up data raises uncertainty about the sustainability of the observed improvements in parental stress and mental wellbeing. Although some evidence suggests that the effects of ACT may be maintained over time (31), the lack of follow-up in this study prevents conclusions about the stability of these benefits. Future studies should address these variables to provide a more comprehensive understanding of the intervention’s effects.

## 6 Conclusion

This study provides preliminary evidence supporting the use of an accessible group-based ACT prating program, delivered flexible via either online videoconferencing or face-to-face formats, as an effective approach in reducing parental stress, general stress and depression, as well as enhancing mental health literacy, subjective wellbeing, and psychological flexibility during the COVID-19 pandemic. Notably, the online format showed as effective as the face-to-face delivery mode in improving parental stress and psychological wellbeing post-intervention. This flexibility in delivery makes ACT particularly valuable during times of social distancing or other disruptions to traditional service provision. However, further research is needed to confirm these results and to address the methodological limitations identified in this study.

## Data Availability

The raw data supporting the conclusions of this article will be made available by the authors, without undue reservation.
